# Computer Algorithms To Detect Bloodstream Infections

**DOI:** 10.3201/eid1009.030978

**Published:** 2004-09

**Authors:** William E. Trick, Brandon M. Zagorski, Jerome I. Tokars, Michael O. Vernon, Sharon F. Welbel, Mary F. Wisniewski, Chesley Richards, Robert A. Weinstein

**Affiliations:** * Centers for Disease Control and Prevention, Atlanta, Georgia, USA;; † Chicago Antimicrobial Resistance Project, Chicago, Illinois, USA;; ‡ Cook County Hospital, Chicago, Illinois, USA;; § Rush Medical College, Chicago, Illinois, USA

**Keywords:** surveillance, bloodstream infection, information system, computer data processing, algorithms, infection control, central-venous catheter, research

## Abstract

We compared manual and computer-assisted bloodstream infection surveillance for adult inpatients at two hospitals. We identified hospital-acquired, primary, central-venous catheter (CVC)-associated bloodstream infections by using five methods: retrospective, manual record review by investigators; prospective, manual review by infection control professionals; positive blood culture plus manual CVC determination; computer algorithms; and computer algorithms and manual CVC determination. We calculated sensitivity, specificity, predictive values, plus the kappa statistic (κ) between investigator review and other methods, and we correlated infection rates for seven units. The κ value was 0.37 for infection control review, 0.48 for positive blood culture plus manual CVC determination, 0.49 for computer algorithm, and 0.73 for computer algorithm plus manual CVC determination. Unit-specific infection rates, per 1,000 patient days, were 1.0–12.5 by investigator review and 1.4–10.2 by computer algorithm (correlation r = 0.91, p = 0.004). Automated bloodstream infection surveillance with electronic data is an accurate alternative to surveillance with manually collected data.

## Abstract 2

Automated bloodstream infection surveillance using electronic data is an accurate alternative to surveillance using manually collected data.

Central-venous catheter (CVC)-associated bloodstream infections are common adverse events in healthcare facilities, affecting approximately 80,000 intensive-care unit patients in the United States each year (*1**,**2*). These infections are a leading cause of death in the United States (*3*) and are also associated with substantially increased disease and economic cost (*4*).

As part of an overall prevention and control strategy, the Centers for Disease Control and Prevention's (CDC) Healthcare Infection Control Practices Advisory Committee recommends ongoing surveillance for bloodstream infection (*2*). However, traditional surveillance methods are dependent on manual collection of clinical data from the medical record, clinical laboratory, and pharmacy by trained infection control professionals. This approach is time-consuming and costly and focuses infection control resources on counting rather than preventing infections. In addition, applying CDC case definitions requires considerable clinical judgment (*5*), and these definitions may be inconsistently applied. Further, human case finding can lack sensitivity (*6*), and interinstitutional variability in surveillance techniques complicates interhospital comparisons (*7*). With the increasing availability of electronic data originating from clinical care (e.g., microbiology results and medication orders), alternative approaches to adverse event detection have been proposed (*8*) and hold promise for improving detection of bloodstream infections. We present the results of an evaluation study comparing traditional, manual surveillance methods to alternative methods with available clinical electronic data and computer algorithms to identify bloodstream infections.

## Methods

The study was conducted at two institutions, both of which participate in the Chicago Antimicrobial Resistance Project: Cook County Hospital, a 600-bed public teaching hospital and Provident Hospital, a 120-bed community hospital. As part of the project, we created a data warehouse by using data from the admission and discharge, pharmacy, microbiology, clinical laboratory, and radiology department databases (*9*). The data warehouse is a relational database that allows us to link data for individual patients from these separate departments. Data are downloaded from the various departmental databases to our warehouse once every 24 hours; therefore, the algorithms can be applied to clinical data from the previous day.

Facility-specific procedures exist for acquiring and processing blood specimens. At both hospitals, the decision to obtain blood cultures was determined solely by medical providers, without input from infection control professionals or study investigators. After CVC removal, the decision to send a distal segment of the CVC for culture was at the discretion of the medical care provider; both microbiology laboratories accepted these specimens for culture. Since considerable interfacility variability likely exists in CVC culture practices beyond Cook County and Provident Hospitals, we decided not to incorporate these culture results into our computer algorithms.

Blood cultures were obtained and processed at Cook County and Provident Hospitals by using similar methods. At Cook County Hospital, blood cultures were obtained by resident physicians or medical students. At Provident Hospital, blood cultures were obtained by phlebotomists outside of the intensive-care units and by a nurse or physician in the intensive-care unit. At each hospital, blood cultures were injected into Bactec (Becton Dickinson, Inc., Sparks, MD) bottles and incubated for up to 5 days in an automated blood culture detection system. When microbial growth was detected, samples were spread onto solid media and incubated overnight.

Using data from several sources, we compiled a list of all patients who had a positive blood culture hospitalized on inpatient units other than the pediatric or neonatal units from September 1, 2001, through February 28, 2002 (study period). Positive blood cultures obtained <2 days after hospital admission and not evaluated by an infection control professional were excluded. Positive blood cultures obtained within 5 days of the initial positive blood culture were considered as part of the same episode; i.e., these blood cultures were considered polymicrobial infections. At Cook County Hospital, we studied a random sample of positive blood cultures. At Provident Hospital, since a relatively small number of cultures were obtained during the study period, we evaluated all positive blood cultures. Approval was obtained by the local and CDC human participant review boards.

### Investigator Review

We used the CDC definition for primary, CVC-associated, laboratory-confirmed bloodstream infection (*10*). Four study investigators, all of whom had previous experience applying these definitions, performed retrospective medical record reviews. Two investigators independently reviewed each medical record. If there was a judgment disagreement between the two investigators, a third reviewer categorized the blood culture. Investigators were blinded to other investigators' reviews and to determinations made by review and by computer algorithms. To minimize the likelihood of investigator interpretation approximating the computer algorithm, i.e., systematic bias in definition interpretation, the details of the computer algorithms were not disclosed to three of the four reviewers. The reviewer who participated in the construction of the computer algorithms functioned in the same capacity as the other three reviewers (i.e., all four reviewers could participate in the initial or final reviews).

### Infection Control Professional Review

During the study period, infection control professionals at Cook County and Provident Hospitals performed prospective hospitalwide bloodstream infection surveillance using the CDC definitions (*10*). Six infection control professionals submitted data, four at Cook County Hospital and two at Provident Hospital; all were registered nurses and had 10–30 years of infection control experience. All six had attended a 1-day surveillance seminar conducted by CDC personnel and had access to an infection control professional who had attended a CDC-sponsored infection surveillance training course; four were certified in infection control.

At Cook County Hospital, a list of all positive blood culture results was generated by a single person in the microbiology laboratory. Duplicates (i.e., the same species identified within the previous 30 days) were excluded, and the list was distributed to the infection control professionals. For those patients who had not been discharged, the infection control professionals reviewed the medical chart, and if their assessment differed from the medical record documentation, they could discuss the case with the medical team. For patients who had been discharged, only the medical record was reviewed. For polymicrobial cultures (i.e., >1 organism isolated from a blood culture), infection control professionals categorized each isolate. The infection control professionals did not participate in the medical team's ward rounds. At Provident Hospital, the procedures were similar except that the laboratory printed out all positive culture results, and the infection control professional manually excluded duplicate results.

Determinations were recorded on a standardized, scannable form, and the forms were sent to a central location, where they were evaluated for completeness and then scanned into a database. In cases where the infection control professional did not record whether the infection was hospital- or community-acquired, we categorized the infection as hospital-acquired if it was detected >2 days after hospital admission.

### Computer Algorithms

We evaluated several methods to categorize blood cultures. First, we evaluated a simple method that required only a computer report of a positive blood culture recovered >2 days after admission plus manual determination of whether a CVC was present.

**Figure 1:**
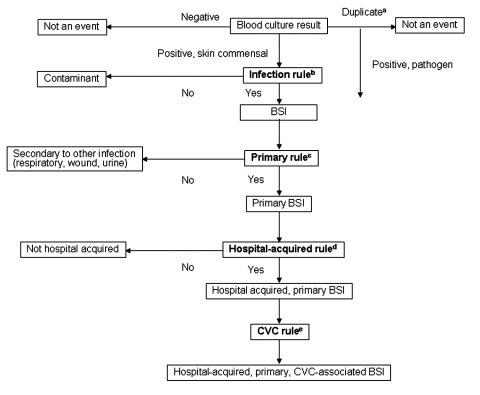
Flowchart displaying the determinations necessary for categorizing positive blood culture by computer algorithm. The rules described in Table 1 are in bold. Blood cultures were obtained from patients at Cook County and Provident Hospitals, September 1, 2001–February 28, 2002, Chicago, Illinois. BSI, blood stream infection; CVC, central-venous catheter. ^a^Same species isolated from blood within 30 days. ^b^Rule B1 or B2 (Table 1). ^c^Rule C1 or C2 (Table 1). ^d^Rule A (Table 1). ^e^Rule D (Table 1).

Second, after consultation with infectious disease clinicians, we developed rules that were combined into more sophisticated computer algorithms (Table 1, Figure 1). Two rules were developed for two of the determinations that were required. For determining infection versus contamination, rule B1 used only microbiology data, while rule B2 used microbiologic and pharmacy data. For determining primary versus secondary (i.e., the organism cultured from the blood is related to an infection at another site) bloodstream infection, rule C1 was limited to a 10-day window, while rule C2 extended throughout the hospitalization. Since two options existed for two separate rules, these rules were combined into four separate algorithms. We report the results of the algorithms that had the best (rules A, B2, C2, and D) and worst (rules A, B1, C1, and D) performance. Consistent with the manual methods, polymicrobial cultures were considered a single event. Polymicrobial blood cultures were considered an infection if any isolate recovered from the same culture met infection criteria, and, in contrast to the manual methods, were considered a secondary bloodstream infection if any isolate that met infection criteria also met criteria to be classified as secondary. Third, since we could not automate CVC detection, we also evaluated augmentation of automated bloodstream infection detection with manual determination of a CVC.

**Table 1:** Computer algorithms and corresponding NNIS system definitions to categorize blood culture isolates, September 2001–February 2002, Cook County Hospital, Chicago, Illinois^a^

Determination^b^	Computer rule	NNIS definitions
Hospital acquired	(A) Acquired blood culture >3 days after hospital admission	No evidence infection present or incubating at time of hospital admission, unless infection was related to previous admission to this hospital
Infection	(B1) Microbiology data: pathogen other than CSC^c^ cultured from blood, or >2 CSC isolates recovered from blood within 5 days of initial positive blood culture	Patient has at least one sign or symptom: fever (>38°C), chills, or hypotension and at least one of the following: pathogen cultured from >1 blood cultures, CSC cultured from >2 blood cultures drawn on separate occasions, CSC cultured from at least 1 blood culture from patient with intravenous line, and physician institutes appropriate antimicrobial drug therapy
	(B2) Microbiology and pharmacy data: pathogen cultured from blood or >2 CSC^d^ isolates within 5 days of initial positive blood culture, or CSC cultured from blood once and vancomycin administered within 3 days before until 1 day after isolate identification	
Secondary bloodstream infection (BSI)^d^	(C1) Time restricted: organism recovered from blood also recovered from a nonblood culture, 3–7 days after the blood culture acquisition date^d^	The organism cultured from the blood is related to an infection at another site
	(C2) Length of stay: organism recovered from blood also recovered from a nonblood culture during the entire length of stay^d^	
Central-venous catheter (CVC) associated^e^	(D) No algorithm developed; all BSI were considered CVC associated	Vascular access device that terminated at or close to heart or one of great vessels within the 48-hour period before BSI developed

^a^NNIS, National Nosocomial Infection Surveillance; CSC, common skin contaminant; BSI, bloodstream infection. ^b^For each determination, if the computer rule or NNIS definition was not met, the isolate was considered as one the following: community acquired, a contaminant, primary BSI, or not CVC associated. ^c^We used the examples of CSCs listed in the NNIS manual: diphtheroids, *Bacillus* spp., *Propionibacterium* spp., coagulase-negative staphylococci, or micrococci. ^d^Catheter tip and stool cultures were excluded for both algorithms. CSCs had to be cultured from a wound for the BSI to be considered as a secondary BSI. ^e^Includes tunneled or nontunneled catheters inserted into the subclavian, jugular, or femoral veins; pulmonary artery catheters; hemodialysis catheter; totally implanted devices (ports); peripherally inserted central catheters; and introducer sheaths.

For Provident Hospital, since the number of positive blood cultures evaluated by each rule was relatively small, we do not report the performance characteristics. We do report the results for the best and worst computer algorithms at each hospital and at both hospitals combined.

### Statistics

For polymicrobial cultures, we analyzed the results at the level of the blood culture. We were primarily interested in evaluating the detection of hospital-acquired, primary, CVC-associated bloodstream infections; therefore, by investigator or infection control professional review, if any isolate from a polymicrobial culture met the necessary criteria, the blood culture was classified as a hospital-acquired, primary, CVC-associated bloodstream infection.

We present the results of comparisons for the blood cultures that were evaluated by all methods. For calculation of sensitivity, specificity, and predictive values, we considered the investigator review to be the reference standard. Next, we calculated the agreement between investigator review and the other methods using the kappa statistic (κ) (*11*). Since all organisms that were not common skin commensals were considered an infection, we included only common skin commensals to evaluate the rule distinguishing infection versus contaminant.

We report bloodstream infection rates per 1,000 patient-days for certain units in the hospital. Hospital units were aggregated according to the type of patient-care delivered, as identified by hospital personnel. For example, data from all nonintensive care medical wards were aggregated. Also, because of the relatively low number of patient-days in the burn, trauma, and neurosurgical intensive-care units (ICU), we aggregated the bloodstream infection rates for these units and report them as specialty ICUs. We calculated the Pearson correlation coefficient for bloodstream infection rates determined by investigator review versus other methods, stratified by hospital unit. Since only a sample of blood cultures was evaluated at Cook County Hospital, the rates were adjusted to account for the unit-specific sampling fraction. We also calculated the Pearson correlation coefficient, comparing the number of bloodstream infections per month identified by investigator review versus the other methods. All analyses were performed by using SAS statistical package version 8.02 (SAS Institute Inc., Cary, NC).

## Results

**Figure 2:**
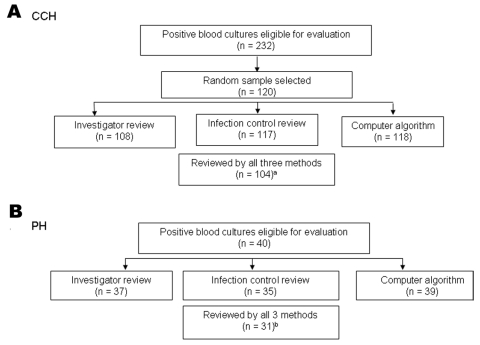
Flowchart displaying the number of blood cultures eligible for evaluation and the number evaluated by investigator review, infection control professional review, and computer algorithm at A) Cook County Hospital (CCH) and B) Provident Hospital (PH), September 1, 2001–February 28, 2002, Chicago, Illinois. ^a^At CCH, 12 medical records were unavailable for investigator review; three positive blood cultures were not evaluated by an infection control professional; and two positive blood cultures did not have culture dates stored electronically and, thus, were inaccessible to the computer algorithm. ^b^At PH, three medical records were unavailable for investigator review, five positive blood cultures were not evaluated by an infection control professional, and one positive blood culture was not in the data warehouse (this blood culture isolate also was not documented in the medical record).

At Cook County Hospital, 104 positive blood cultures from 99 patients were evaluated by all methods (Figure 2A). Of the 99 patients, most were male (58%) and were cared for in non-ICUs (65%); the median patient age was 52 years. Of the 104 patients with positive blood cultures, 83 (79%) were determined to have infection by investigator review, 55 (53%) had primary bloodstream infection, and 39 (37.5%) had hospital-acquired, primary, CVC-associated bloodstream infection. The most common organisms were coagulase-negative staphylococci (n = 45), *Staphylococcus aureus* (n = 23), *Enterococcus* spp. (n = 11), *Pseudomonas aeruginosa* (n = 4), *Escherichia coli* (n = 4), and *Candida albicans* (n = 4); nine (8.7%) infections were polymicrobial.

At Provident Hospital, 40 positive blood cultures were eligible for investigator review; 31 cultures from 28 patients were evaluated by all methods (Figure 2B). Of the 28 patients, most were male (54%) and cared for in non-ICUs (68%); the median patient age was 60 years. Of the 31 patients whose cultures were evaluated by all methods, 29 (94%) were determined to have infection by investigator review, 17 (55%) were primary, and 9 (29%) were hospital-acquired, primary CVC-associated bloodstream infection. The most common organisms were *S. aureus* (n = 9), coagulase-negative staphylococci (n = 6), *Enterococcus* spp. (n = 5), *P. aeruginosa* (n = 3), *E. coli* (n = 2), and *C. albicans* (n = 2); no polymicrobial infections occurred.

### Hospital versus Community-acquired Rule

When we evaluated the hospital versus community-acquired rule at Cook County Hospital, the computer rule A had a slightly higher sensitivity, specificity, and κ statistic than did the infection control professional review (Table 2). Only one computer rule was evaluated (Table 1).

**Table 2:** Positive blood cultures as categorized by computer rules or infection control professional (ICP) review, compared to investigator review,^a^ Cook County Hospital, Chicago, IL

Determination	Method	No. cultures^c^	Sensitivity (%)	Specificity (%)	κ
Hospital vs. community acquisition	Computer rule A	77	97	73	0.74
	ICP review	77	94	67	0.62
Infection vs. contamination^d^	Computer rule B2^e^	43	77	71	0.49
	ICP review	43	77	76	0.53
Primary vs. secondary	Computer rule C2^f^	76	90	57	0.49
	ICP review	76	83	64	0.48

^a^Our reference standard. ^b^The single best rule for each determination is displayed. ^c^Since all determinations were not made for each blood culture, e.g., contaminants often were not further categorized, the number of cultures evaluated varies. ^d^Infection determination is presented for common skin commensals only; other organisms were considered as infections; see Table 1 for definitions. ^e^Rule B2 evaluated microbiology and pharmacy results; see Table 1. ^f^Rule C2 evaluated microbiology results during the entire length of stay; see Table 1.

### Infection versus Contamination Rule for Common Skin Commensals

At Cook County Hospital, infection control professional review and computer rule B2 (which used microbiologic and pharmacy data) had similar performance (Table 2). Computer rule B1 (which used only microbiologic data) was less sensitive (55%) but had a similar κ (0.45).

### Primary versus Secondary Rule

At Cook County Hospital, infection control professional review and computer rule C2 had similar sensitivities, specificities, and κ statistics. Both determinations had limited specificity, i.e., some secondary infections were misclassified as primary bloodstream infections. The 12 infection syndromes classified as primary by computer algorithm and secondary by investigator review were lower respiratory tract (n = 5, 42%), intraabdominal (n = 3, 25%), skin or soft tissue (n = 2, 17%), or surgical site (n = 2, 17%); no urinary tract infection was misclassified as a primary bloodstream infection by computer algorithm. Computer rule C1, which evaluated only culture results within a time frame around the blood culture acquisition date, had lower specificity than rule C2 (data not shown).

### Bloodstream Infection Algorithm

For overall ability to detect hospital-acquired, primary, CVC-associated bloodstream infection, we found that the simplest method (computer determination of a positive culture plus manual CVC determination) performed better than infection control professional review (κ = 0.48 vs. κ = 0.37, Table 3). The best and worst performing computer algorithms had good performance (κ = 0.49 and κ = 0.42, respectively). When manual determination of a CVC was added to the best performing computer algorithm, the correlation was significantly better than the infection control professional review (κ = 0.73, p = 0.002). At each hospital, the best performing computer algorithm, with or without manual CVC determination, performed better than infection control professional review. For both hospitals combined, the number of hospital-acquired, primary, CVC-associated bloodstream infections varied by method, investigator review (n = 48), infection control professional review (n = 56), positive culture plus manual CVC determination (n = 86), computer algorithm (n = 64), and computer algorithm plus manual CVC determination (n = 48).

**Table 3:** Comparing alternative methods for determining if positive blood cultures represented a hospital-acquired, primary, central-venous catheter–associated bloodstream infection, Cook County and Provident Hospitals, Chicago, Illinois^a^

Method	% sensitivity	% specificity	% PVP	% PVN	κ (95% CI)
Cook County Hospital (n = 104)					
Investigator review (reference method)	–	–	–	–	–
Infection control professional review	67	75	62	79	0.41 (0.24–0.59)
Positive blood culture + CVC determination^b^	100	55	57	100	0.48 (0.35–0.62)
Worst computer algorithm (rules A, B1, C1, D)^c^	72	74	62	81	0.44 (0.27–0.62)
Best computer algorithm (rules A, B2, C2, D)^d^	79	72	63	85	0.49 (0.33–0.66)
Computer algorithm + CVC determination^b^	79	88	79	88	0.67 (0.52–0.82)^e^
Provident Hospital (n = 31)					
Investigator review (reference method)	–	–	–	–	–
Infection control professional review	56	68	42	79	0.22 (–0.13–0.56)
Positive blood culture + CVC determination^b^	100	59	50	100	0.46 (0.20–0.70)
Worst computer algorithm (rules A, B1, C1, D)^c^	78	64	53	88	0.35 (0.04–0.65)
Best computer algorithm (rules A, B2, C2, D)^d^	89	68	53	94	0.48 (0.19–0.76)
Computer algorithm + CVC determination^b^	89	95	89	95	0.84 (0.63–1.0)^e^
Summary for both hospitals (n = 135)					
Investigator review (reference method)	–	–	–	–	–
Infection control professional review	65	74	57	79	0.37 (0.21–0.53)^e^
Positive blood culture + CVC determination^b^	100	56	56	100	0.48 (0.36–0.60)
Worst computer algorithm (rules A, B1, C1, D)^c^	72	74	62	81	0.42 (0.27–0.57)
Best computer algorithm (rules A, B2, C2, D)^d^	81	72	62	87	0.49 (0.35–0.63)
Computer algorithm + CVC determination^b^	81	90	81	90	0.73 (0.61–0.85)^e^

^a^PVP, predictive value positive; PVN, predictive value negative, CI, confidence interval. ^b^Presence of a central-venous catheter (CVC) determined by investigator medical record review. ^c^The computer algorithm with the worst agreement, which used only microbiology data for the determination of infection vs. contaminant (rule B1, Table 1), and an abbreviated time period for the determination of primary vs. secondary (rule C1, Table 1). ^d^The computer algorithm with the best agreement, which used the microbiology and pharmacy data for the determination of infection vs. contaminant (rule B2, Table 1), and the entire length of stay for the determination of primary vs. secondary (rule C2, Table 1). ^e^Agreement between investigator review and the best performing computer algorithm plus CVC determination was significantly better than between investigator and infection control professional reviews, i.e., p value < 0.05.

### Comparison of the Monthly Variation

**Figure 3:**
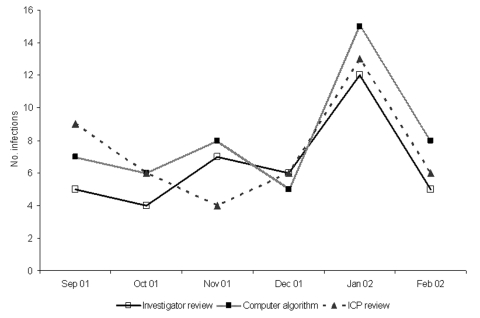
Display of the monthly number of hospital-acquired, primary, central-venous catheter–associated bloodstream infections (BSIs) determined by separate methods, and correlation of the computer algorithm and infection control professional (ICP) review to the investigator review, Cook County Hospital, Chicago, Illinois. Computer algorithm r = 0.89, p = 0.02; ICP review r = 0.71, p = 0.11.

At Cook County Hospital, when the number of hospital-acquired, CVC-associated bloodstream infections per month was considered, infection control professional review (r = 0.71) was not as well correlated with investigator review as the computer algorithm (r = 0.89) was (Figure 3). When we augmented the computer algorithm with manual CVC determination, the effect was minimal on the correlation between the monthly variations (data not shown). At Provident Hospital, the monthly number of bloodstream infections was too small to provide meaningful comparisons.

### Comparisons of Unit-Specific Bloodstream Infection Rates

**Figure 4:**
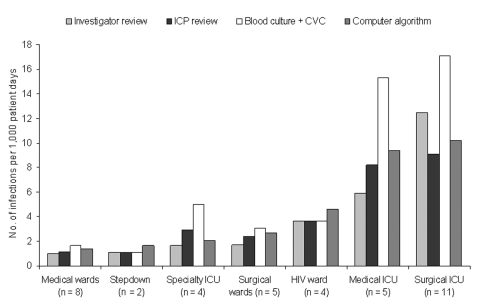
Comparison of the hospital-acquired, primary, central-venous catheter (CVC)-associated bloodstream infection (BSI) rate for adult patient–care units determined by two separate manual methods (i.e., infection control professional [ICP] and investigator review), by positive blood culture plus manual CVC determination, and by computer algorithm, Cook County Hospital, September 1, 2001–February 28, 2002, Chicago, Illinois. The number of hospital-acquired, primary, CVC-associated bloodstream infections determined by investigator review is displayed in parentheses. Correlation coefficient (r) and p value for comparisons between investigator review and each method were as follows: infection control professional review r = 0.95, p = 0.001; blood culture + central line determination r = 0.90, p = 0.006; computer algorithm r = 0.91, p = 0.004. ICU, intensive-care unit.

At Cook County Hospital, the patient care unit–specific bloodstream infection rates determined by investigator review versus those determined by computer algorithm had the same rank from highest to lowest: surgical intensive care, medical intensive care, HIV ward, surgical wards, specialty intensive care, step-down units, and medical wards (Figure 4). The bloodstream infection rates were well correlated between the investigator review and the computer algorithm or infection control professional review. At Provident Hospital, the bloodstream infection rates per 1,000 patient days were as follows: on the non-ICUs, investigator review = 0.41, infection control professional review = 0.39, and computer algorithm = 0.62; in the ICUs, investigator review = 2.05, infection control professional review = 3.68, and computer algorithm = 3.68.

## Discussion

We used electronic data from clinical information systems to evaluate the accuracy of computer algorithms to detect hospital-acquired primary CVC-associated bloodstream infections. Compared with investigator chart review (our reference standard), we found that computer algorithms that used electronic clinical data outperformed manual review by infection control professionals. When the computer algorithm was augmented by manually determining whether a CVC was present, agreement with investigator review was excellent. These results suggest that automated surveillance for CVC-associated bloodstream infections by using electronic data from clinical information systems could supplement or even supplant manual surveillance, which would allow infection control professionals to focus on other surveillance activities or prevention interventions.

CDC's National Nosocomial Infection Surveillance (NNIS) system reports CVC-associated, hospital-acquired, primary bloodstream infection rates. Determining whether a bloodstream infections is primary and catheter-associated is worthwhile because some prevention strategies differ for catheter-associated versus secondary bacteremias; e.g., the former can be prevented through proper catheter insertion, maintenance, and dressing care (*2**,**12*). However, hospitalwide bloodstream infection surveillance at the three Cook County Bureau of Health Services hospitals is labor-intensive and estimated to consume, at a minimum, 452 person hours per year (*9*). This estimate is low because it does not include the time required to identify and list bacteremic patients or record these patients into an electronic database.

Automated infection detection has several advantages, including the following: applying definitions consistently across healthcare facilities and over time, thus avoiding variations among infection control professionals' methods for case-finding and interpretations of the definitions; freeing infection control professionals' time to perform prevention activities; and expanding surveillance to non-ICUs, where CVCs are now common (*13*).

Since positive blood culture results are central to the bloodstream infection definition and readily available electronically, adapting the bloodstream infection definition is relatively easy for computer algorithms. For other infection syndromes (e.g., hospital-acquired pneumonia), the rules may be more difficult to construct. Despite the relative simplicity of bloodstream infection algorithms, many determinations, or "rules," had to be considered, and various options were considered for each.

The rule for determining hospital versus community acquisition, i.e., a positive blood culture >3 days after admission, performed well at Cook County Hospital but poorly at Provident Hospital (data not shown), where some community-acquired bloodstream infections were not detected until >3 days after hospital admission. Since some of these positive blood cultures were caused by secondary bloodstream infections, these delays did not adversely affect the performance of the final algorithm, which incorporated additional rules.

The computer rule for determining primary versus secondary bloodstream infection was problematic when the presumed source of these bloodstream infections was not culture-positive, usually for lower respiratory tract infections. We minimized this problem by evaluating nonblood culture results during a patient's length of stay; however, this solution would not be desirable for patient populations with prolonged lengths of stay. The specificity of automated primary bloodstream infection detection could be improved by interpreting radiology reports or using International Classification of Disease codes to automate pneumonia detection (*14*).

To determine infection versus contamination for common skin commensals by including appropriate antimicrobial use for single positive blood cultures as a criterion for bloodstream infection, we may be evaluating physician prescribing behavior rather than identifying true bloodstream infections; i.e., some episodes of common skin commensals isolated only once are contaminants unnecessarily treated with antimicrobial drugs (*15*). Since the CDC's bloodstream infection definition includes this criterion, including antimicrobial use in our computer infection rule improved the performance of this algorithm. Despite the potential inaccuracy, reporting the frequency of antimicrobial drug therapy for common skin commensals isolated only once may help healthcare facilities identify episodes of unnecessary drug therapy.

Other investigators have tried to either fully automate infection detection or automate identification of patients who have a high probability of being infected (*16**–**19*). These studies demonstrate the feasibility of automated infection detection. Our study adds additional information by comparing a fully automated computer algorithm, a partially automated computer algorithm (including manual CVC determination), and infection control professional blood culture categorization to the investigators' manual evaluation.

Our study has several limitations. Investigator review may have been influenced by knowledge of the computer algorithms; however, three of the four reviewers were not familiar with the details of the computer algorithms. In addition, our evaluation included only patients at a public community hospital or public teaching hospital, and our findings may not be generalizable to other healthcare facilities. In particular, several factors could influence the performance characteristics of both computer algorithm and manual surveillance, including the frequency of blood culture acquisition, CVC use, the distribution of pathogens, and the proportion of bloodstream infections categorized as secondary. Also, we expected better agreement between investigator and infection control professional reviews. Potentially, agreement could be improved by additional infection control professional training. The computer algorithm could also likely be improved by incrementally refining the algorithm or including additional clinical information. The cost of refining the algorithm with local data or including more clinical data would be a decrease in the generalizability or feasibility of the algorithms. Further, many hospital information systems have not been structured so that adverse event detection can be automated. The algorithms we used could be improved when hospital information systems evolve to routinely capture additional clinical data (e.g., patient vital signs) or process and interpret textual reports (e.g., radiograph reports) (*14**,**20**,**21*).

Reporting data to public health agencies electronically has recently become more common (*22**,**23*). One important and achievable patient safety initiative is reducing CVC-associated bloodstream infections (*24*). Traditional surveillance methods are too labor intensive to allow hospitalwide surveillance; therefore, NNIS has recommended focusing surveillance on ICUs. However, intravascular device use has changed and, currently, most CVCs may be outside ICUs (*13*). Using electronic data holds promise for identifying some infection syndromes, and hospitalwide surveillance may be feasible. Hospital information system vendors can play a key role in facilitating automated healthcare-associated adverse event detection. Our study demonstrates that to detect hospital-acquired primary CVC-associated bloodstream infections, using computer algorithms to interpret blood culture results was as reliable as a separate manual review. These findings justify efforts to modify surveillance systems to fully or partially automate bloodstream infection detection.
